# Preclinical Assessment in Juvenile Sheep of an Allogeneic Bone Tissue Engineering Product with Wharton’s Jelly Mesenchymal Stromal Cells

**DOI:** 10.3390/cells14120862

**Published:** 2025-06-07

**Authors:** Raquel Cabrera-Pérez, Irene Carreras-Sánchez, Ángela Roig-Molina, Alba López-Fernández, Irene Portas-Torres, Laura Batlle-Morera, Roberto Vélez, Joaquim Vives

**Affiliations:** 1 Bioprocessing for Advanced Cell Therapies (BACT) Group, Cell Therapy Service, Blood and Tissue Bank (BST), 08005 Barcelona, Catalonia, Spain; 2 Musculoskeletal Tissue Engineering Group, Vall d’Hebron Research Institute (VHIR) and Universitat Autònoma de Barcelona (UAB), 08035 Barcelona, Catalonia, Spain; 3 Tissue Engineering Unit, Centre for Genomic Regulation (CRG), Barcelona Biomedical Research Park (PRBB), 08003 Barcelona, Catalonia, Spain; 4 Musculoskeletal Tumour Unit and Orthopaedic Surgery Department, Vall d’Hebron University Hospital (HUVH), 08035 Barcelona, Catalonia, Spain; 5 Medicine Department, Universitat Autònoma de Barcelona (UAB), 08193 Bellaterra, Catalonia, Spain

**Keywords:** advanced therapy medicinal product, bone regeneration, fibrin, synthetic bone substitute, multipotent mesenchymal stromal cells, Wharton’s jelly

## Abstract

Secondary osteonecrosis (ON) is a common complication in paediatric cancer survivors. Combining multipotent mesenchymal stromal cells (MSCs) with core decompression surgery halts disease progression and stimulates bone regeneration. However, the success of advanced therapy medicinal products (ATMPs) requires versatile “off-the-shelf” tissue engineering products (TEPs). This study evaluated the safety and efficacy of TEPs loaded with allogeneic MSCs from Wharton’s jelly (WJ-MSCs) in a large-animal model of bone regeneration to support a paediatric investigational plan for ON patients. WJ-MSC-laden fibrin-based hydrogels combined with a synthetic bone substitute (PRO-DENSE^TM^) were tested in 16 juvenile sheep (8 males and 8 females) distributed in four experimental groups. Each animal received four cylindrical bone defects in the femoral and tibial epiphyses and was assessed at 6 and 12 weeks. Safety was confirmed, and bone regeneration was observed across all groups. A combination of WJ-MSCs with PRO-DENSE^TM^ led to improved histological scores, osteogenesis, and construct integration. Trabecular bone volume also increased more in cellular groups over time. However, effects were inconsistent across groups, reflecting the variability seen in clinical trials and highlighting the significant impact of factors such as immunogenetic compatibility, MSC batch potency, and interaction with the recipient’s microenvironment on the therapeutic effectiveness and successful clinical translation of allogeneic ATMPs.

## 1. Introduction

Almost 400,000 cases of cancer are diagnosed annually worldwide in children and adolescents under 19 years old. Among the different types of cancer, leukaemias (28.8%), lymphomas (13.8%), and central nervous system tumours (11%) are the most frequent [1]. For paediatric leukaemias and lymphomas, 5-year survival rates are around 80–90%, according to the American Cancer Society [2]. However, the incidence of secondary late effects associated with cancer treatments is higher in immature organs and tissues and contributes to a high burden of morbidity in adults who were treated for cancer during childhood. These patients usually develop severe or life-threatening complications and chronic health conditions, including bone tissue degeneration [3,4].

One of the current treatment options for aggressive haematological tumours in paediatric patients is haematopoietic stem cell transplantation (HSCT). In HSCT, bone marrow (BM) progenitor cells are removed by high-dose chemotherapy and radiotherapy and then replaced with healthy autologous or allogeneic BM cells, mobilised peripheral blood, or umbilical cord blood [5]. Although these procedures have led to a marked increase in survival, new risks and complications affecting the mid- to long-term quality of life (QoL) of patients arise [6]. The most prevalent adverse effect associated with cytotoxic treatments and HSCT is secondary osteonecrosis (ON), which can compromise the integrity of different bones and joints and results in significant morbidity. High-dose corticosteroids, immunosuppressive therapy, and radiation used in conditioning regimens impair bone vascularisation and cellular viability, leading to ischemia and bone necrosis. In the paediatric population, the incidence of ON ranges between 4% and 44% due to skeletal immaturity and continuous bone growth [7,8]. Today, ON treatments are based on slowing down disease progression and stimulating mechanisms of bone regeneration. Among these therapeutic options, core decompression is usually proposed as a salvage method to avoid joint replacement in patients with early-stage disease. This procedure can be performed alone or in combination with multipotent mesenchymal stromal cells (MSCs) or BM implantation and cancellous bone or autologous chondrocyte grafting. Additionally, pharmacological treatments targeting presumed pathophysiological mechanisms that may be implicated in ON development (such as low-molecular-weight heparin, prostacyclin analogues, statins, and bisphosphonates) and non-surgical therapies (including hyperbaric oxygenation, extracorporeal shockwave treatment, and single-pulsed electromagnetic field therapy) have also been explored [8].

MSCs, a specific type of stem cell with the capacity to generate bone tissue, are considered a promising tool in cell therapy and tissue engineering for the treatment of bone diseases, including the early stages of ON, either as a cellular suspension or loaded onto the surface of biomaterials or devitalised bony scaffolds [9,10]. Among the different tissue sources for the isolation of MSCs, autologous BM has been the most frequently used, and their wide application in preclinical studies and current experimental therapies has confirmed their excellent safety profile [11,12,13]. However, harvesting autologous MSCs from BM is associated with comorbidities, a delay in the administration of cells due to the time required to expand cells up to sufficient numbers *ex vivo*, and the risk of using cells isolated from patients with systemic diseases or who have received drugs that could affect cell viability and/or functionality, thus compromising their therapeutic potential [14,15]. To overcome this situation and guarantee the success of novel advanced therapy approaches, versatile, “off-the-shelf” osteogenic tissue engineering products (TEPs) based on allogeneic substances of human origin (SoHOs) must be developed in line with specific regulatory pathways [16,17]. In this context, the treatment of ON with allogeneic BM-MSCs has been previously explored by our group in a preclinical ovine model of ON of the femoral head (ONFH) with promising results, demonstrating that the osteogenic capacity of allogeneic BM-MSCs is equivalent to that of autologous BM-MSCs in sheep [18]. However, BM-MSC-based therapies present significant shortcomings that limit their allogeneic use, including limited availability and potential ineligibility due to donor age, clinical history, and the accumulation of somatic mutations. More recently, Wharton’s jelly-derived MSCs (WJ-MSCs) have emerged as an attractive alternative to MSCs derived from adult tissues in allogeneic clinical applications due to: i) their accessibility, ease of expansion *in vitro*, safety profile, multipotentiality, and biological activity to modulate the immune system; and ii) they are subjected to minimal ethical considerations and come from healthy, young, and same-age donors, thereby reducing inter-batch variability [19,20,21]. Importantly, our latest published study showed that WJ-MSCs promote new bone formation when administered into the bone microenvironment in immunodeficient mice [22].

Based on the aforementioned advantages, and after demonstrating the bone-forming capacity of WJ-MSCs *in vivo*, we proposed a novel tissue engineering approach for bone regeneration consisting of the combination of synthetic biomaterials and osteogenic hydrogels made of allogeneic WJ-MSCs in young patients. More specifically, our main objective was to determine the safety and efficacy of a TEP combining a WJ-MSC-laden fibrin-based hydrogel with a synthetic calcium phosphate/sulphate biomaterial (PRO-DENSE^TM^) in a juvenile ovine model of cylindrical bone defects, with a focus on supporting a paediatric investigational plan from a regulatory perspective. Importantly, as the model used is not specific to osteonecrosis but rather represents a general bone defect, the results may be extrapolated to a broader range of clinical conditions requiring bone regeneration.

## 2. Materials and Methods

### 2.1. Genetic Modification of Ovine WJ-MSCs

WJ-MSCs were isolated from ovine umbilical cord tissue as previously described [23] and genetically modified to express the mCherry reporter protein. To this end, 10^6^ WJ-MSCs were electroporated using the NEPA21 electroporator system (Nepa Gene Co., Ltd., Ichikawa, Chiba Prefecture, Japan) with 5 µg of the pPB-CAG-mCherry-IRES-PURO plasmid (Addgene, Cambridge, MA, USA) in combination with 3 µg of the pPBase vector (Addgene, Cambridge, MA, USA) in order to randomly integrate the construct into the genome. Next, transfected cells were selected with 0.5 µg/mL puromycin (A11138-03, Gibco, Waltham, MA, USA) and expanded *in vitro* up to sufficient numbers using expansion media composed of Dulbecco’s Modified Eagle’s Medium (DMEM, Gibco, Waltham, MA, USA) supplemented with 10% ovine pooled serum. All cultures were maintained at 37 °C and 5% CO_2_ in humidified incubators, and media were changed every 3–4 days. Cell number and viability were determined by the haemocytometer-based trypan blue dye exclusion assay.

### 2.2. Characterisation of mCherry-Labelled WJ-MSCs

The immunophenotypic characterisation of ovine WJ-MSCs was performed by fluorescence-activated cell sorting (FACS) using the following antibodies: mouse anti-sheep CD31-fluorescein isothiocyanate (CD31-FITC; Bio-Rad Laboratories, Inc., Hercules, CA, USA); mouse anti-sheep CD44-fluorescein isothiocyanate (CD44-FITC; Bio-Rad Laboratories, Inc., Hercules, CA, USA); mouse anti-human CD166-allophycocyanin (CD166-APC; Miltenyi Biotec, Bergisch Gladbach, Germany); and mouse anti-sheep MHC class II DR monomorphic-fluorescein isothiocyanate (MHCII-FITC; Bio-Rad Laboratories, Inc., Hercules, CA, USA). Cells were stained for 15 min at room temperature (RT) and then washed and resuspended in phosphate-buffered saline (PBS; Gibco, Waltham, MA, USA). Acquisition was carried out using a CytoFLEX (Beckman Coulter, Inc., Brea, CA, USA), and data were analysed with Kaluza v2.2 (Beckman Coulter, Inc., Brea, CA, USA) software.

The differentiation capacity of mCherry-labelled ovine WJ-MSCs into adipo-, chondro-, and osteogenic lineages was determined *in vitro.* Differentiation media composed of the StemPro adipogenesis differentiation kit (Gibco, Waltham, MA, USA), StemPro chondrogenesis differentiation kit (Gibco, Waltham, MA, USA), and StemPro osteogenesis differentiation kit (Gibco, Waltham, MA, USA), supplemented with 100 units/mL of penicillin and 100 µg/mL streptomycin (Penicillin-Streptomycin; Sigma-Aldrich, Burlington, MA, USA), were used for adipo-, chondro-, and osteogenic induction, respectively. MSC cultures were maintained at 37 °C and 5% CO_2_ in humidified incubators, and media were changed every 3–4 days. Red Oil O (Sigma-Aldrich, Burlington, MA, USA), Safranin O (Sigma-Aldrich, Burlington, MA, USA), and Alizarin Red (Merck Millipore, Burlington, MA, USA) staining were performed after 32 days to determine the outcome of the adipo-, chondro-, and osteogenic differentiation.

### 2.3. In Vitro Assessment of Biocompatibility of mCherry-WJ-MSCs with Synthetic Scaffolds

Prior to the implantation of TEPs in sheep, biocompatibility in terms of cell survival and cell proliferation of mCherry-labelled ovine WJ-MSCs in combination with synthetic scaffolds consisting of a fibrin-based hydrogel composed of 0.5 mL of plasmalyte saline solution (Baxter International Inc., Deerfield, Chicago, IL, USA) containing 2% *w*/*v* of human serum albumin (HSA; Grifols, S.A., Sant Cugat del Vallès, Spain), 0.5 mL of 1% *w*/*v* hyaluronic acid (sodium hyaluronate; BioIberica S.A.U., Esplugues de Llobregat, Spain) in PBS (Lonza, Basel, Switzerland), 0.5 mL of fibrinogen (Tisseel; Baxter International Inc., Deerfield, Chicago, IL, USA), 75 µL of thrombin (Tisseel; Baxter International Inc., Deerfield, Chicago, IL, USA), and a calcium phosphate/sulphate biomaterial (PRO-DENSE^TM^; Wright Medical Group N.V., Amsterdam, The Netherlands) was assessed *in vitro*. To this end, 3 × 10^6^ MSCs were resuspended in 0.5 mL of plasmalyte containing 2% (*w*/*v*) of HSA. Next, 0.5 mL of 1% (*w*/*v*) hyaluronic acid in PBS and 0.5 mL of fibrinogen were sequentially added and mixed by pipetting. This resulted in 1.5 mL of the cellular mixture, which was then transferred to a well of a non-adherent 24-well plate. Finally, 75 μL of thrombin was added and mixed with a sterile pipette tip. Prior to clot formation, a hand-generated PRO-DENSE^TM^ disc was introduced into the mixture. Cellular TEPs were maintained in expansion media at 37 °C and 5% CO_2_ in humidified incubators up to 7 days, and media were changed every 3–4 days.

To determine the presence, morphology, distribution, status, and survival of MSCs, live/dead staining (Live/Dead Viability/Cytotoxicity Kit; Invitrogen, Carlsbad, CA, USA) was performed immediately after the generation of TEPs and after 3 and 7 days of culture. The results were evaluated with a fluorescence inverted microscope (DMIL FLUO; Leica, Wetzlar, Germany).

### 2.4. Animal Selection and Ethics Statement

The preclinical study was performed in 11- to 14-month-old healthy and skeletally immature sheep (*Ovis aries*) of the Ripollesa × Lacaune breed (*n* = 16; 8 males and 8 females) weighing 43.25 ± 9.27 kg and supplied by A.M. ANIMALIA, S.L. (Girona, Spain). Prior to the inclusion of any animal in the study, an X-ray examination was performed to confirm skeletal immaturity based on the radiolucent appearance of epiphyseal growth plates in the proximal tibia. Once selected, animals were transported to the animal core facility of the Vall d’Hebron Research Institute (VHIR), where they were housed after an acclimatisation period of at least one week. They were kept following a 12 h light/dark schedule and provided with a standard diet and water ad libitum. All animal procedures were approved by the animal experimentation ethics committee of the VHIR (protocol number 75.19) and performed by specialised personnel in accordance with national, regional, and European legislation (Royal Decree (RD) 53/2013, Decree 214/97, and Directive 2010/63/EU, respectively) to guarantee the animals’ welfare.

### 2.5. Experimental Design and Study Groups

For the *in vivo* experiments, a total of 16 sheep were included and distributed in 4 experimental groups (4 sheep per group, including 2 males and 2 females) (see Table 1). Four cylindrical bone defects were generated per animal: one in the distal femoral epiphysis and one in the proximal tibial epiphysis of both hind limbs. As a result, 64 cylindrical bone defects were created and treated as follows: 4 animals (16 defects) were treated with a synthetic calcium phosphate/sulphate biomaterial and an acellular fibrin-based hydrogel (group 1); 4 animals (16 defects) were treated with a synthetic calcium phosphate/sulphate biomaterial and a WJ-MSC-laden fibrin-based hydrogel (group 2); 4 animals (16 defects) were treated with an acellular fibrin-based hydrogel (group 3); and 4 animals (16 defects) were treated with a WJ-MSC-laden fibrin-based hydrogel (group 4).

### 2.6. Sedation, Anaesthesia, and Analgesia

Prior to the surgical intervention, animals were kept under food deprivation for 16 to 20 h. On the day of the surgery, animals were first sedated with 0.5 mg/kg of midazolam (Midazolam Sala, Lab. Ramon Sala S.L., Sant Joan Despí, Spain) by intramuscular (IM) injection. A fentanyl transdermal patch (2.5–5 μg/kg Fendivia^®^, Nycomed, Zürich, Switzerland) was then applied on the forelimb and kept for 72 h. The cephalic vein was canalised for the extraction of 6 mL of peripheral blood to obtain baseline haematological and biochemical data, as well as for the administration of 4 mg/kg of propofol (Propofol Fresenius, Fresenius Kabi AG, Bad Homburg, Germany) to induce anaesthesia. Additionally, the middle articular artery was also canalised in order to monitor the arterial pressure. During these canalisations, oxygen was administered using an anaesthetic mask. Once in the operating room, tracheal intubation was performed, and inhalational anaesthesia was maintained by mechanical ventilation with a 50–60% inspired oxygen fraction and 2% isoflurane (Forane^®^, AbbVie Farmacéutica, S.L.U., Madrid, Spain). An orogastric tube was also introduced through the mouth of the animal until the stomach to avoid ruminant bloat and minimise the accumulation of free gases in the rumen. During the surgical procedure, Ringer’s lactate solution (Viaflo Hartmann, Baxter International Inc., Deerfield, Chicago, IL, USA) as well as fentanyl (Fentanest^®^, Kern Pharma S.L., Terrassa, Spain) were continuously infused at 2–3 mL/kg/h and 10 mg/kg/h, respectively, and different parameters such as temperature, heart rate, arterial pressure, inspired/expired fractions of oxygen/isoflurane, CO_2_ arterial pressure, and final CO_2_ concentration were continually monitored and recorded. As antibiotic prophylaxis, animals received 22 mg/kg of intravenous cefazolin (Cefazolina Sala, Lb. Ramon Sala S.L., Sant Joan Despí, Spain) at the time of anaesthetic induction and 15 mg/kg of IM amoxicillin (Duphamox LA, Zoetis Inc., Parsippany, NJ, USA) in the postoperative period.

### 2.7. Surgical Procedures

In the first step, the area of intervention in the femorotibial joint of the corresponding hind limb was shaved and cleaned with a povidone-based soap and water. The area was then dried and sprayed with a povidone iodine alcoholic solution. During the surgical interventions, intra-operative X-rays of the femorotibial joint were performed in anteroposterior and lateral projections to precisely determine the locations for the generation of the cylindrical bone defects in the distal medial epiphysis of the femur and the proximal medial epiphysis of the tibia. An incision of approximately 1 cm was performed in the location where the defects were created, and a trephine (bone biopsy trephine 8 mm external diameter, 170 mm long, Synthes Holding AG, Zuchwill, Switzerland) was introduced 2 cm deep. The surrounding periosteum was meticulously eliminated. Bone cylinders were then extracted, and the generated defects (8 mm diameter × 20 mm depth) were filled with the corresponding TEP. In animals belonging to experimental groups 3 and 4, the bone defect was filled with a mixture containing the fibrin-based hydrogel with or without WJ-MSCs. Similarly, in animals included in experimental groups 1 and 2, the bone defect was first filled with a mixture containing the fibrin-based hydrogel with or without cells, and then, after coagulation of the TEP, approximately 1 cm^3^ of PRO-DENSE^TM^ biomaterial (Wright Medical Group N.V., Amsterdam, The Netherlands) was injected. Finally, the surgical incisions were closed via layers of Vicryl 3.0 (Ethicon, Johnson&Johnson, New Brunswick, NJ, USA), aluminium was sprayed over the area of intervention to avoid infection, and an X-ray was performed to confirm the correct locations and treatment of the cylindrical bone defects. For all experimental groups, the right hind limb was treated first. Six weeks later, a second intervention was carried out in the left hind limb following the same procedure.

### 2.8. TEP Manufacturing

For the generation of cellular TEPs, 3 × 10^6^ mCherry-labelled ovine WJ-MSCs were resuspended in 0.5 mL of plasmalyte (Baxter International Inc., Deerfield, Chicago, IL, USA) containing 2% (*w*/*v*) of HSA (Grifols, S.A., Sant Cugat del Vallès, Spain) and drawn into a 3 mL syringe. Once in the operating room and after creation of the cylindrical bone defects, the syringe containing the cellular suspension was connected to a 3-way Luer lock valve. An additional syringe containing 0.5 mL of 1% (*w*/*v*) hyaluronic acid (BioIberica S.A.U., Esplugues de Llobregat, Spain) in PBS was also connected to the 3-way valve, and the contents of both syringes were mixed carefully, avoiding the formation of bubbles. After mixing, one of the syringes was discarded, replaced by a third syringe containing 0.5 mL of fibrinogen (Tisseel; Baxter International Inc., Deerfield, Chicago, IL, USA), and mixed again. Finally, the syringe containing the fibrinogen was replaced by a 1 mL syringe containing 75 µL of thrombin (Tisseel; Baxter International Inc., Deerfield, Chicago, IL, USA); the solutions were then mixed and allowed to rest for 1–2 min. At this point, the cellular hydrogel was used to immediately fill the bone defect. For acellular TEPs, 0.5 mL of plasmalyte (Baxter International Inc., Deerfield, Chicago, IL, USA) containing 2% (*w*/*v*) of HSA (Grifols, S.A., Sant Cugat del Vallès, Spain) was used.

The same procedure was performed in the case of TEPs containing the calcium phosphate/sulphate biomaterial (PRO-DENSE^TM^; Wright Medical Group N.V., Amsterdam, The Netherlands). PRO-DENSE^TM^ was then prepared following the manufacturer’s instructions, and approximately 1 cm^3^ of biomaterial was injected into the bone defect containing the cellular or acellular hydrogel.

Sterile conditions were maintained throughout the entire process.

### 2.9. Postoperative Clinical Monitoring

From surgical intervention until the day of euthanasia, sheep were clinically evaluated to detect any evidence of pain or immune reactions to the treatments. Additionally, animals were weighed regularly to record weight changes, and blood samples were taken for the routine analysis of haematological and biochemical parameters at different time points as follows: before each surgical intervention (week 0 and week 6); 15 days after the surgeries (week 2 and week 8); and at the end of the experimental period (week 12).

### 2.10. Euthanasia, Necropsy, and Sample Processing

Twelve weeks after the first surgical intervention in the right hind limb, the animals were sedated by an IM injection of midazolam (0.5 mg/kg) (Midazolam Sala, Lab. Ramon Sala S.L., Sant Joan Despí, Spain) and later euthanised with an intravenous overdose of sodium thiopental (50–60 mg/kg) (Tiobarbital Braun 1 g, B. Braun Medical S.A, Melsungen, Germany). Both hind limbs were then harvested, and femurs and tibias were carefully extracted. After performing X-ray and micro-CT imaging analysis, treated bones were sectioned 1–2 cm proximally and distally for each of the cylindrical defects and processed for histological and immunohistochemical assays.

After the animals were euthanised, a macroscopic necropsy of the most representative organs (heart, lung, liver, spleen, kidney, and gonads) was carefully performed in an orderly, complete, and systematic manner in 4 animals treated with cellular hydrogels (2 from group 2 and 2 from group 4) and in 2 animals treated with acellular hydrogel (group 3) in order to detect malignant transformations associated with the use of WJ-MSCs. Additionally, representative samples were taken from the gonads of these animals to rule out the presence of WJ-MSCs in this tissue.

### 2.11. Micro-Computed Tomography (micro-CT)

The day of the euthanasia, all harvested femurs and tibias were scanned with a desktop micro-CT imaging system (Quantum FX micro-CT Imaging System, Perkin Elmer, Waltham, MA, USA). Acquisition parameters were 90 kV and 200 µA, with a 40 mm field of view. The regenerated bone volume (mm^3^) in the cylindrical bone defects was measured using the AMIDE (Amide’s a Medical Image Data Examiner) software as follows: in the case of femurs, two different regions of interests (ROIs) were defined, one containing the cortical area and one containing the trabecular area of the bone (in both cases, the diameter of the cylindrical ROI was set at 8 mm and the depth was adjusted depending on the anatomical characteristics of each animal, with a maximum total depth of 20 mm (cortical + trabecular ROI)); for tibias, a single cylindrical ROI was established containing the cortical area (a diameter of 8 mm and the depth was adjusted for each animal). Since the thickness of the trabecular bone contained between the epiphyseal plate and the medullary cavity in the tibia is less than in the femur, most of the defects were placed in the medullary cavity, and only bone regeneration in the cortical bone could be determined. In samples from groups 1 and 2, the PRO-DENSE^TM^ volume was subtracted from the total volume based on the grey intensity, which was higher in the biomaterial than in bone.

### 2.12. Histological Studies

Bone sections containing the treated cylindrical defects were fixed in 4% buffered formalin (VWR International, LLC, Radnor, PA, USA) for 1 week and then decalcified in Decalcifier I solution (Leica, Wetzlar, Germany), with a solution change every 3–4 days. After one month, each bone defect was divided into two halves, and the decalcification process was continued for approximately two weeks until paraffin embedding was performed. Then, 5 µm thick sections were obtained, stained with haematoxylin and eosin (H&E), and observed under a light microscope. The histological sections obtained after this process were of poor quality, and their tissue architecture was compromised.

In an attempt to improve the quality of the sections, histological blocks were deparaffinised and samples were decalcified again in Decalcifier I solution (samples from cellular groups 2 and 4) or nitric acid (samples from acellular groups 1 and 3) with a solution change every 3–4 days until a sponge-like consistency was obtained (between 3 and 10 months, depending on the sample). At this time, paraffin embedding was performed again, and 5 µm thick sections were obtained, stained with H&E, and observed under a light microscope. After this second decalcification step, the quality of the histological sections improved. However, evaluable bone sections could not be obtained for all samples. In those samples in which the defect area could be identified, bone regeneration was scored semi-quantitatively according to the parameters specified in Supplementary Table S1. The result for each sample corresponds to the mean calculated from the score given by three independent members involved in the project after previous anonymisation and randomisation of the samples.

### 2.13. Immunohistochemical Staining for the Detection of mCherry-Labelled WJ-MSCs

Immunohistochemical detection of mCherry-labelled WJ-MSCs was performed in 5 µm thick sections obtained from samples of animals treated with cellular TEPs (experimental groups 2 and 4). Due to the aforementioned problems in the decalcification process, only 8 samples could be analysed (6 samples from group 2 and 2 samples from group 4). Briefly, sections were rehydrated in a series of xylene and ethanol baths, antigen retrieval was performed with a 10 mM sodium citrate solution, and endogenous peroxidase activity was inhibited by treatment with 3% hydrogen peroxide. After the washing and permeabilisation steps, blocking of non-specific antigens was performed with a 2.5% BSA solution. Samples were then incubated with the following primary and secondary antibodies at dilutions of 1:250 and 1:500, respectively: rabbit polyclonal anti-mCherry antibody (NBP2-25157, Novus Biologicals, LLC, Centennial, CO, USA) and goat anti-rabbit IgG (HRP) (31460, ThermoFisher Scientific Inc., Waltham, MA, USA). Localisation of the secondary HRP-conjugated antibody was revealed using the ABC Peroxidase Staining Kit (32020, ThermoFisher Scientific Inc., Waltham, MA, USA) and eBioscience™ DAB Advanced Chromogenic Kit (8801-4965-72, ThermoFisher Scientific Inc., Waltham, MA, USA). Finally, slides were counterstained with Mayer’s Haematoxylin (51275, Sigma-Aldrich, Burlington, MA, USA), dehydrated in a series of ethanol and xylene baths, mounted using a DPX mountant for histology (06522, Sigma-Aldrich, Burlington, MA, USA), and visualised under a light microscope. A cellular TEP composed of a WJ-MSC-laden fibrin-based hydrogel (group 4) and histological sections from a sheep treated with an acellular hydrogel (group 3) were used as positive and negative controls, respectively.

### 2.14. Biodistribution Assays

At the endpoint, the presence/absence of mCherry-labelled WJ-MSCs in gonads from animals of cellular groups (experimental groups 2 and 4) was assessed by conventional PCR. Genomic DNA was purified from frozen ovaries/testicles using the QIAmp DNA Mini Kit (51304, QIAGEN N.V., Venlo, The Netherlands) according to the manufacturer’s instructions and quantified by spectrophotometry using NanoDrop Lite (Thermo Scientific Inc., Waltham, MA, USA). mCherry amplicon was determined using a total amount of 100 ng of genomic DNA using the following primers: 5′-CCAAGCTGAAGGTGACCAA-3′ (forward) and 5′-TCTTCTTCTGCATTACGGGG-3′ (reverse). The expected band size after 37 cycles of amplification was 288 bp. The results were visualised in a 1% agarose gel stained with SYBR green using the LaunchDoc software.

### 2.15. Statistical Analysis

Graphs and statistical analyses were performed using GraphPad Prism 5 (GraphPad Software, Inc., La Jolla, CA, USA). Data representation and statistical tests used in each case are specified in the corresponding figure legend. Statistical significance was set at *p* < 0.05.

## 3. Results

### 3.1. Characterisation of mCherry-WJ-MSCs and Biocompatibility with the Proposed TEPs

**Figure 1:**
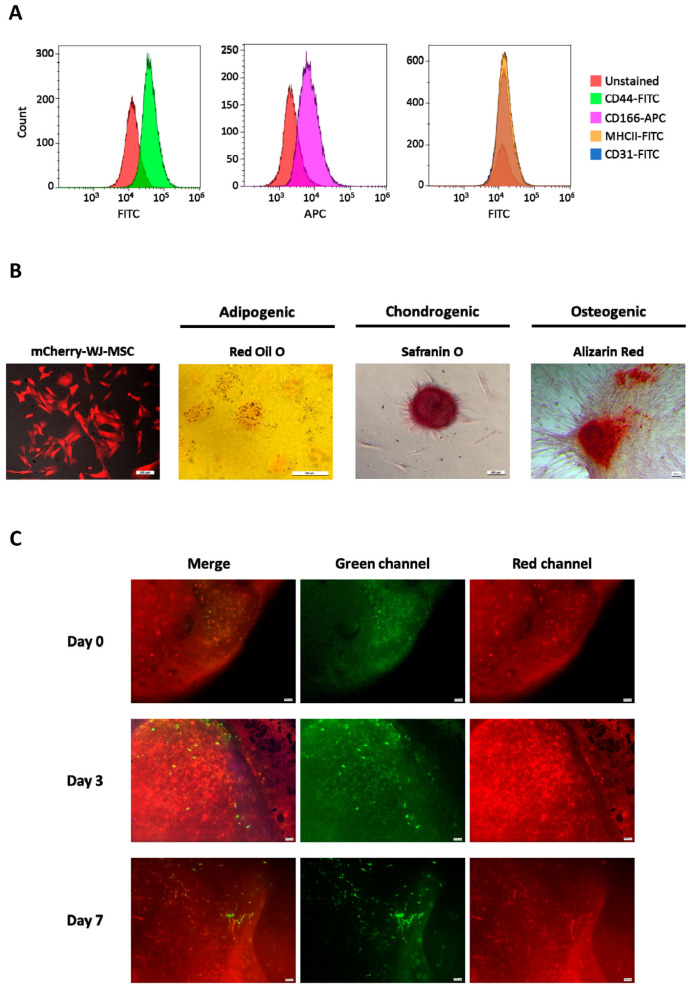
Characterisation of mCherry-WJ-MSCs and biocompatibility with synthetic scaffolds. (**A**) Phenotypic characterisation of mCherry-labelled WJ-MSCs showing the expression of positive (CD44 and CD166) and negative (CD31 and MHCII) surface markers. (**B**) Representative images showing the appearance of mCherry-WJ-MSC cultures and their trilineage differentiation potential. Scale bars: 100 µm. (**C**) Representative images of live/dead staining performed on mCherry-WJ-MSCs combined with synthetic scaffolds at days 0, 3, and 7. Merged images, as well as split green and red fluorescence channels, are shown. Scale bars: 100 µm.

In order to obtain labelled WJ-MSCs to allow cell tracking after injection, WJ-MSCs isolated from ovine umbilical cords were genetically modified by electroporation to express the mCherry reporter. After gene editing, 97.2% of the cellular population was positive for mCherry expression (97.2% average expression; range: 96.7–97.6%; *n* = 5). mCherry-labelled WJ-MSCs were characterised to ensure that random integration of the mCherry construct in the cell genome did not disturb the critical quality attributes (CQAs) of MSCs. mCherry-WJ-MSCs were comparable to wild-type MSCs, displaying similar growth rates (µ_max_) of 0.310 ± 0.085 and 0.350 ± 0.127 days^−1^; doubling times of 2.320 ± 0.608 and 2.130 ± 0.764 days; and cumulative population doublings (CPDs) of 4.125 ± 0.643 and 5.960 ± 1.513, respectively. The biological characteristics of MSCs were also maintained in genetically engineered MSCs. Specifically, they were able to grow in adherence, presented fibroblastic morphology, and were positive for the expression of CD44 and CD166 surface markers, while the expression of CD31 and MHCII markers remained negative (Figure 1A). Moreover, the *in vitro* differentiation potential of mCherry-WJ-MSCs into the adipogenic, chondrogenic, and osteogenic lineages was also confirmed (Figure 1B).

Prior to their use *in vivo*, the biocompatibility of mCherry-WJ-MSCs in combination with the synthetic scaffolds was assessed *in vitro* by live/dead staining. Due to the high intensity of the red fluorescence provided by the mCherry reporter, discrimination between live (green) and dead (red) cells was difficult (Figure 1C, Merge). However, the observation of cellular TEPs in the green fluorescent channel revealed a high rate of live cells colonising the scaffold surface, especially at day 3 (Figure 1C, green channel). Additionally, when comparing the state of the cells at day 0 with that at days 3 and 7, a clear transition from rounded to fibroblastic morphology was also observed (Figure 1C), confirming the biocompatibility of the different components of the TEP.

### 3.2. Clinical Results

In order to evaluate the safety and efficacy of the proposed TEPs, a total of 16 sheep were included in the study and distributed in four experimental groups. Fifteen out of sixteen animals completed the study. One female treated with acellular hydrogel (group 3) died for unknown reasons 4 days prior to the expected euthanasia. The macroscopic necropsy ruled out any relationship between the death and the experimental treatment. Therefore, as the death occurred a few days before the established endpoint, bone samples were taken, analysed, and included in the study. Importantly, no alterations or malignant transformations associated with either the use of WJ-MSCs or with the synthetic scaffolds were detected in any of the macroscopic necropsies performed (Supplementary Table S2).

The surgical procedures were determined to be feasible and reproducible. In only 3 out of a total of 64 cases, the bone defect broke through the bone, generating a cylindrical channel of 8 mm in diameter in the femur (1 sheep) or the tibia (2 sheep) in the affected animals. In one sheep, the femoral synovial capsule was damaged during the creation of the bone defect in both surgical interventions, probably due to the abnormal anatomy of the animal (Supplementary Table S2). These incidents did not affect the recovery of the animals in any case. In general terms, all sheep evolved favourably after surgeries, and only a mild transient limp was observed within the first 48 h after treatment in animals of all experimental groups. No significant changes in body temperature or signs of infection or inflammation near the implantation areas were found 6 weeks after treatment.

Regarding blood tests, elevated liver enzymes (alanine aminotransferase [ALT] and gamma-glutamyl transferase [GGT]) were observed in one sheep from group 3 and one from group 4 from the 6th week of treatment. However, these findings were not attributed to the treatment. For the rest of the parameters analysed, values fell within the physiological range for all sheep throughout the study period, and no differences were observed between groups.

Finally, no differences in absolute body weight or weight gain were observed between groups (Supplementary Table S3).

### 3.3. Bone Regeneration Capacity of the Synthetic Scaffolds

**Figure 2:**
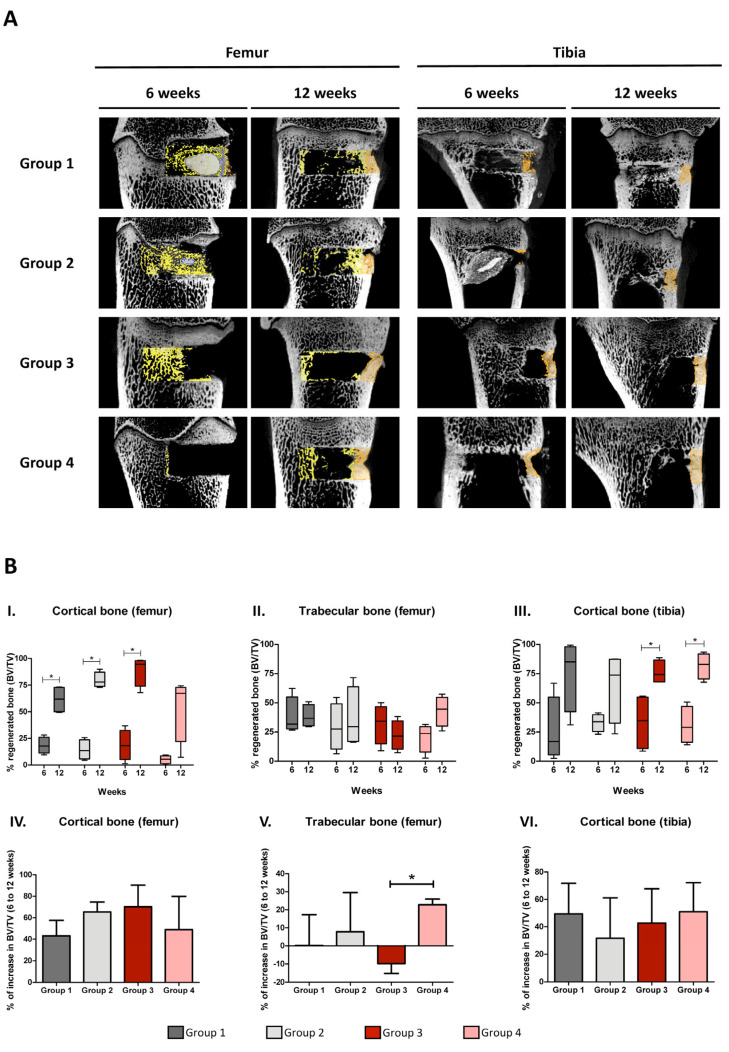
Micro-computed tomography analysis. (**A**) Representative images of micro-CT scans showing regenerated areas of cortical bone (orange), trabecular bone (yellow), and PRO-DENSE^TM^ (blue). (**B**) Quantification of cortical and trabecular bone regeneration in the defect area in femur (graphs I and II) and tibia (graph III) at 6 and 12 weeks after treatment. Whiskers indicate Min to Max values. Increment of bone regenerated area from 6 to 12 weeks (graphs IV, V, and VI). Bars represent mean ± SEM. Statistical significance (*) was set at *p* < 0.05 (Mann–Whitney test). BV/TV, bone volume/total volume in the defect area.

The bone regeneration capacity of the different TEPs was evaluated after 6 and 12 weeks of treatment by means of micro-CT scanning and histological H&E staining. For the analysis of micro-CT images, two different ROIs were defined, containing the cortical or the trabecular area of the bone defect. However, since the thickness of trabecular bone contained between the epiphyseal plate and the medullary cavity in the tibia is less than in the femur, most of the tibia defects were placed in the medullary cavity. Therefore, bone regeneration in the tibia defects was only determined in the cortical bone (Figure 2A).

Based on the micro-CT results, no differences between treatments were found when comparing the percentage of the area occupied by regenerated bone (% of bone volume (BV)/total volume (TV)) in either the femur (cortical and trabecular bone) or the tibia (cortical bone) for any of the experimental time points (Figure 2). However, the effect of time was critical to observe an increase in the volume of new cortical bone in all study groups.

Six weeks after treatment, the mean percentage of regenerated cortical bone volume ranged from 5.1% to 18.6% when considering all the defects created in the femur (*n* = 16), and from 25.7% to 33.5% in the case of the tibia (*n* = 16) (Figure 2B, graphs I and III and Supplementary Figure S1). Six weeks later, these values increased, ranging from 54.0% to 88.8% in the femur and from 64.6% to 81.7% in the tibia (Figure 2B, graphs I and III and Supplementary Figure S1A). This represents an increment of between 43.2% and 70.2% in the femur, and between 31.6% and 51.0% in the tibia (Figure 2B, graphs IV and VI). Volumes of regenerated cortical bone were higher in the tibia than in the femur at 6 weeks (Supplementary Figure S1A). However, these differences were only significant in group 4, most likely due to intragroup variability.

In contrast, in the case of femur trabecular bone, no significant increase in the percentage of regenerated bone volume was observed from 6 to 12 weeks for any study group (Figure 2B, graph II). Nevertheless, as shown in graph V of Figure 2B, cellular groups showed a higher increase in the percentage of regenerated trabecular bone over time (group 2: 7.77 ± 21.78; group 4: 22.79 ± 3.15) than acellular groups (group 1: 0.3 ± 17; group 3: −9.76 ± 5.38). In fact, a significant difference was observed when comparing groups 3 and 4.

Regarding sex, cortical bone regeneration tended to be higher in males than in females for both the femur and tibia at 6 and 12 weeks after treatment. However, significant differences were only found between sexes after 12 weeks when considering all the defects created in the femur and tibia of all experimental treatment groups (*n* = 16; Supplementary Figure S1B).

**Figure 3:**
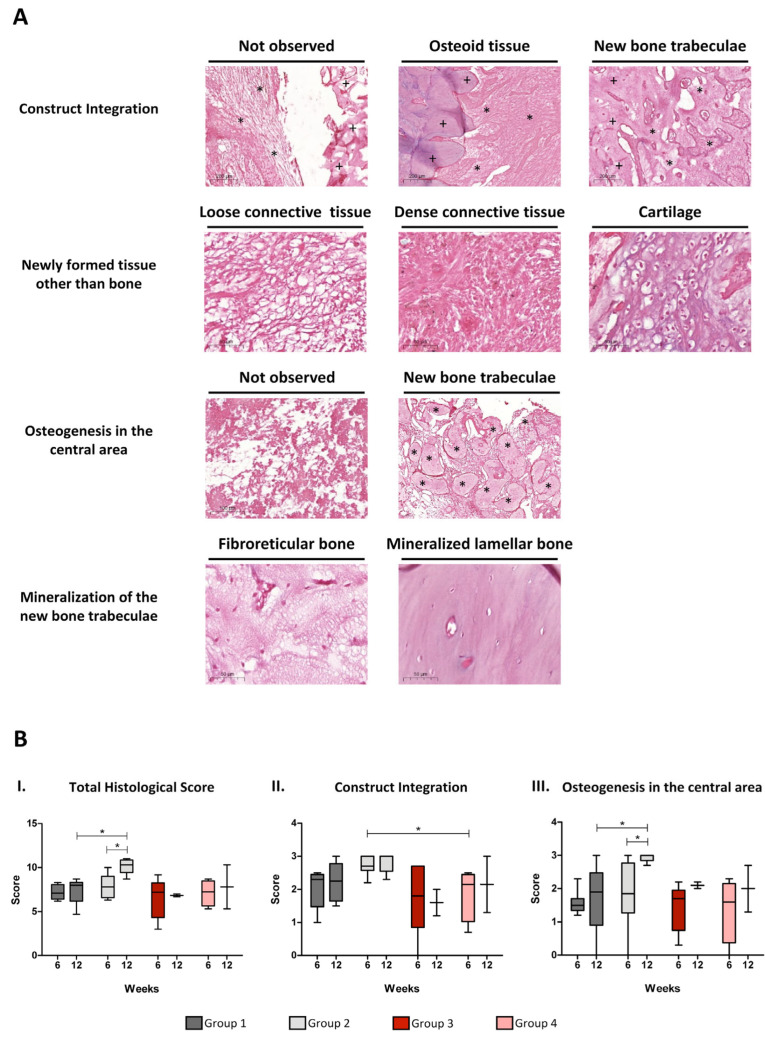
Histological assessment of bone regeneration. (**A**) Representative images of haematoxylin/eosin staining showing the tissue structures evaluated according to the parameters defined in Supplementary Table S1. For construct integration, pre-existing bone (+) and construct (*) are indicated. For osteogenesis in the central area, new bone trabeculae are also marked (*). Scale bars are defined in all images. (**B**) Semi-quantitative results of bone regeneration. Total histological score (graph I) and significant parameters (construct integration, graph II; osteogenesis in the central area, graph III) are shown. Whiskers indicate Min to Max values. Statistical significance (*) was set at *p* < 0.05 (Mann–Whitney test).

Histological results were semi-quantitatively evaluated based on the parameters and scores defined in Supplementary Table S1. Representative images of the tissue structures found in histological H&E staining are shown in Figure 3A. Following this methodology, the TEP providing a major degree of bone regeneration was the one combining a WJ-MSC-laden fibrin-based hydrogel with a synthetic calcium phosphate/sulphate biomaterial (group 2). In fact, a significant difference in the total histological score was observed when comparing groups 1 and 2 after 12 weeks of treatment. Regarding the effect of time, group 2 was also the only one showing a significant increase in the total histological score from 6 to 12 weeks (Figure 3B, graph I).

Among the parameters contributing to the total histological score, significant differences between treatments were only observed for construct integration and osteogenesis in the central area. Specifically, construct integration was superior in group 2 in comparison with group 4 at 6 weeks (Figure 3B, graph II), and osteogenesis in the central area was more abundant in group 2 in relation to group 1 after 12 weeks of treatment (Figure 3B, graph III).

### 3.4. Persistence of mCherry-WJ-MSCs in the Defect Site and Gonads

**Figure 4:**
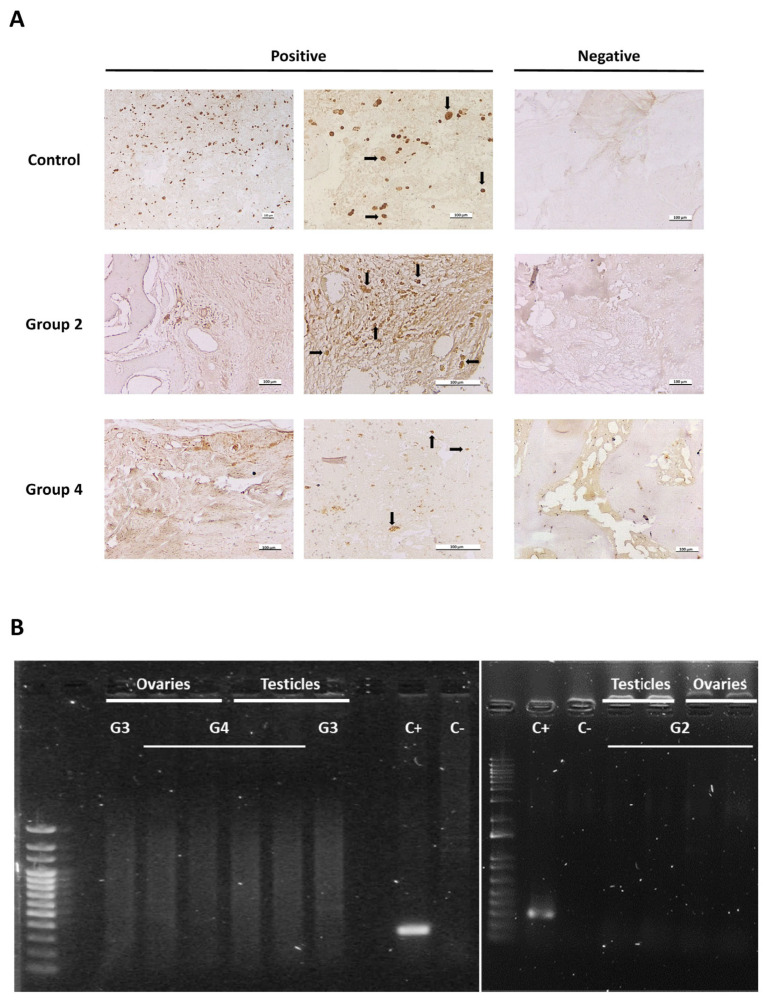
Persistence of mCherry-WJ-MSCs in the defect site and gonads. (**A**) Representative images of mCherry immunostaining in histological sections of mCherry-WJ-MSC-laden fibrin-based hydrogel (control) and 6-week-treated bones (group 2 and group 4). Black arrows indicate positive cells. Scale bars: 100 µm. (**B**) Results of PCR amplification of the mCherry sequence in the gonads of animals treated with cellular TEPs (group 2 (G2) and group 4 (G4)). DNA from mCherry-WJ-MSC cultures (C+), water (C−), and DNA from the gonads of a sheep treated with acellular TEP (group 3 (G3)) were included as controls. Band size: 288 pb. DNA ladder: BrightMAX™ 100–2000 bp (L0015, Canvax).

Since WJ-MSCs were labelled, the persistence of allogeneic mCherry-WJ-MSCs in the defect site was evaluated after 6 and 12 weeks of treatment by immunohistochemical detection of the mCherry protein in histological sections of sheep from cellular groups (experimental groups 2 and 4). Eight specimens were further used for histological processing of the samples (see Materials and Methods), as follows: six from group 2 (four samples after 6 weeks and two samples after 12 weeks of treatment) and two from group 4 (both samples after 6 weeks of treatment). Additionally, an mCherry-WJ-MSC-laden fibrin-based hydrogel and two samples from one sheep in group 3 (one sample after 6 weeks and one sample after 12 weeks of treatment) were also included as positive and negative controls, respectively. Representative images of the results obtained are shown in Figure 4A.

After 6 weeks of treatment, allogeneic mCherry-WJ-MSCs were detected in the site of the defect in three out of six samples (one sample from group 2 and two samples from group 4). In contrast, 12 weeks after treatment, we were unable to find the administered cells in either of the two samples included in this study.

Apart from the macroscopic necropsies performed in animals treated with cellular TEPs, the presence of mCherry-WJ-MSCs in the gonads was also examined in testicles and ovaries obtained from sheep in groups 2 and 4 to obtain additional data regarding the safety of the proposed therapies. As shown in Figure 4B, amplification of the mCherry sequence was not detected in any case.

## 4. Discussion

Osteonecrosis is caused by a variety of factors, including trauma, corticosteroid use, autoimmune diseases, chemotherapy, radiotherapy, and hematologic disorders. Secondary ON is a well-recognised, common, and debilitating therapy-related complication in patients with aggressive childhood haematological tumours that significantly reduces QoL in paediatric cancer survivors. Although optimal treatment is yet to be determined, cell therapy based on the use of cultured osteoblasts, MSCs, or BM concentrate in combination with core decompression surgery has been proposed to halt disease progression and stimulate bone regeneration. Following these strategies, encouraging results have been obtained in preclinical and clinical studies [10,24,25,26]. However, there is a lack of data supporting their use in paediatric patients. Indeed, all applications for marketing authorisation for new medicines have to include the results of studies as described in an agreed paediatric investigation plan (PIP), which is a development programme aimed at ensuring that the necessary data are obtained through studies in children to support the authorisation of a medicine for paediatric use. Unlike general toxicology studies in young adult and adult animals, an assessment of toxicity in juvenile animals requires the evaluation of direct effects, as well as an evaluation of any effects on sexual maturation, growth, and potential long-term effects [27]. In bone tissue regenerative approaches in particular, Malhotra et al. showed that the rate of bone growth in femoral and proximal tibia defects of 8, 11, and 14 mm in diameter was higher in skeletally immature (18-month-old) sheep compared with aged, skeletally mature (5-year-old) animals [28].

In our study, we tested a novel allogeneic therapy consisting of osteogenic TEPs combining WJ-MSC-laden fibrin-based hydrogels with a synthetic calcium phosphate/sulphate biomaterial (PRO-DENSE^TM^) in an ovine model of cylindrical bone defects. To this end, ovine mCherry-labelled WJ-MSCs were used. Prior to TEP implantation, *in vitro* assays confirmed that the genetic modification of the cells did not alter MSC attributes and demonstrated the biocompatibility between the cells and the different components of the TEPs. After 6 and 12 weeks of treatment, evidence of bone regeneration was found in all study groups according to micro-CT results and histopathological assessment in both the cortical and trabecular ROIs of femurs and tibiae from male and female sheep. However, when comparing the effect of using cellular or acellular TEPs with or without PRO-DENSE^TM^, no robust significant differences were observed in the outcome of new bone formation.

MSCs are well known to display biological features of potential relevance for regenerative therapy, including their multipotency, anti-inflammatory, and immunomodulatory properties. Most *in vitro* and many *in vivo* studies published in the literature have suggested that MSCs have the potential to increase osteoinduction and osteogenesis, thus promoting bone regeneration by cell autonomous osteogenic commitment, paracrine signalling to recruit other cell types, and modulation of the inflammatory phase of tissue regeneration [9,13,29,30,31]. Compared to permissive MSCs from other tissue sources, WJ-MSCs are considered to be recalcitrant to make bone tissue in a timely and efficient manner [32,33,34,35,36]. Nevertheless, they present unique characteristics that make them ideal candidates for the development of allogeneic “off-the-shelf” solutions for reducing manufacturing time and improving production sustainability. Furthermore, recent studies carried out by our group demonstrated that WJ-MSCs promote bone formation in immunodeficient mice comparable to BM-MSCs [22].

Based on the results obtained in our study, we cannot conclude that cellular TEPs provide a significant clinical benefit for bone regeneration. Nonetheless, we found evidence supporting a positive effect associated with the use of MSCs, given that (i) micro-CT results revealed that the increment in trabecular bone volume from 6 to 12 weeks was higher in cellular groups and (ii) the total histological score as well as the osteogenesis in the central area was significantly increased in group 2 in comparison with group 1 after 12 weeks of treatment. There are different factors that could influence and limit the osteogenic capacity of MSCs in sheep, namely, (i) the microenvironment in which the cells have been administered; (ii) the competent immunological status of the sheep; and (iii) the osteogenic potency of the selected WJ-MSC batch and its interaction with the microenvironment provided by the recipient sheep.

In our previous study in mice, we performed instillation of WJ-MSCs into the medullary cavity of the tibiae and therefore directly into an intra-bony environment, which provides an exceptional osteogenic and osteoinductive niche surrounded by a native osteoconductive scaffold [22]. However, intra-bony injection is not a common practice in clinics, where most approaches require the filling of critical or considerably sized defects. In the present study, cells were embedded into a fibrin matrix, and there was no direct contact with the intraosseous stroma until resorption of the construct matrix and formation of new blood vessels occurred. As evidenced by previous findings of our group, the secretome of BM-MSCs contains soluble osteoinductive factors that could be essential to ensure a successful outcome with WJ-MSCs [37]. The identification of these essential factors and their addition to the TEP formulation (alone or in combination with angiogenic molecules) could be critical to mimic the bone microenvironment, thus ensuring and potentiating a clinical benefit when using WJ-MSCs for bone regeneration. Regarding osteoconductivity, we decided to incorporate PRO-DENSE^TM^ into the TEP formulation. The results obtained demonstrated that the presence of this bone substitute, which could be indispensable in providing enough mechanical stability and avoiding bone collapse in critical defects located in anatomical areas requiring high load bearing, does not alter the bone regeneration capacity of the TEP. In fact, a higher degree of construct integration was observed when comparing group 2 with group 4 after 6 weeks.

Regarding the immunological status of the animals, immunodeficient mice are characterised by possessing a defective innate immunity and a lack of mature T cells, B cells, and natural killer cells. This condition impairs the detection and removal of foreign cells and allows for their persistence at the injection site, which could also account for long-term effectiveness. The clinical use of MSCs from HLA-mismatched donors has been validated and reported [38]. Although MSCs are described as immunoprivileged and weakly immunogenic since they lack HLA-DR expression, HLA-mismatched MSCs administered to immunocompetent recipients are recognised as foreign and eliminated by different mechanisms of heterologous immunity and tissue rejection, thus impairing cell engraftment in the host tissue [39]. In the field of regenerative therapy, the most accepted hypothesis is that, once homed in the damaged tissue, MSCs activate tissue regeneration by paracrine signalling rather than by direct differentiation into functional tissue cells [40,41]. However, there is also evidence showing their direct implication in the formation of new tissue structures [22,42,43,44]. In view of this, some concerns arise regarding the correlation between the persistence of the administered MSCs and their therapeutic effect. In our study, we were able to detect the infused cells in three out of six analysed samples after 6 weeks of treatment and, although the outcome of bone regeneration in these samples was no better than in those in which mCherry protein was not detected, conclusions cannot be drawn due to the small number of samples analysed. Therefore, in future studies with allogeneic cells, HLA compatibility between donor and recipient should be determined in order to answer this question. If a clear correlation is found, and in order to avoid having to find a matched donor, induced MSCs (iMSCs) obtained from induced pluripotent stem cells (iPSCs) from super-donors whose haplotype matches a high percentage of a given society could be considered [45,46]. Apart from the persistence of the cells, another question to be answered is whether there is any time requirement prior to MSC elimination that might influence the success in the clinical response.

Regarding the osteogenic potency of the therapeutic MSC cell line and its interaction with the recipient, research in the MSC field is focused on identifying markers that could reliably predict the clinical efficacy of a specific MSC batch in a particular group of patients. However, despite scientific endeavours to define homogeneous clinical quality attributes and the mechanism of action of MSCs, there are still some questions that need to be elucidated. This limits the implementation of standardised potency assays that predict the therapeutic efficacy of MSCs and hinders the selection of the optimal MSC batch, establishment of the therapeutic dose, and the choice of administration route, which in turn leads to a lack of consistency in the *in vivo* osteogenesis provided by MSCs and is a critical drawback in the translation of MSC-based experimental therapies to clinical practice [47]. In the context of bone formation, MSCs with higher fibroblast colony-forming unit (CFU-F) efficiencies, a larger proportion of small-sized cells, and enhanced growth capacity have been reported to form increased amounts of bone at ectopic sites. The expression of CD200, STRO-1 and PDGFR-α surface markers, the mRNA levels of *IGF1* and *SGRN*, and the lack of EphB3 signalling have also been defined as potential determinants for the osteogenic commitment of MSCs [48,49,50,51]. However, given that we do not yet have a validated and standardised panel of markers, use of an MSC batch consisting of a pool of MSC lines obtained from different donors could be a strategy to potentiate clinical efficacy. Apart from the genetic and molecular signature of the donor MSCs, individual patients present different underlying biology that accounts for the high intragroup variability that is usually observed in preclinical and clinical trials. Thus, biomarkers and/or immune and disease characteristics must also be considered to determine MSC responsiveness [52,53].

In the present study, male and female sheep were included with the aim of identifying possible sex-associated responses to regenerative medicine treatments. Significant differences between sexes were only found after 12 weeks when considering all the defects created in femurs and tibias from animals of different treatment groups as a whole. However, in line with the results published by other authors in the field, bone regeneration consistently tended to be greater in males than in females [54,55]. A higher sensitivity to sex hormones and thus, ovarian function, has been described as the main cause accounting for this sexually dimorphic response [56,57].

In order to guarantee the principle of the 3Rs, bone defects were created in both the tibia and femur to achieve a larger sample size (N) with a smaller number of animals. The results obtained suggested a greater but not significant degree of regeneration in the tibia compared to the femur, especially in the short term, although faster femoral healing has been described in humans [58,59]. This could be explained by the limited space between the tibial plateau and the intramedullary (IM) canal in sheep, as the IM canal is a direct source of endogenous BM-MSCs with the potential for tissue regeneration [28]. Although MSC implantation in the IM canal could limit cellular confinement at the local site of administration, no adverse events or macroscopic histopathological alterations associated with the use of MSCs were identified in cellular groups. In relation to this, treatment with MSCs from different origins by either local injection or intravenous administration has proven to be safe in clinical trials, and no serious adverse events other than transient fever, administration-site adverse events, constipation, fatigue, and sleeplessness have been reported so far [12].

As with all preclinical studies, our study has some limitations that should be recognised. First, we used healthy sheep. Among the available animal models, sheep represent a valuable model for the testing of translational skeletal therapies, as they are docile animals and similar to humans in body weight, bone anatomy, and mineral composition [60]. However, diseased microenvironments and concomitant treatments in ON patients, as well as variations in density and microstructure between ovine and human bones, must be taken into account [60]. Second, our therapy was mainly focused on the treatment of paediatric populations, and thus, skeletally immature sheep were used. This fact, in combination with the defect size (which was 8 mm in diameter to allow the generation of multiple defects per individual), could diminish significant differences between groups, as age and defect volume are both crucial factors determining the intrinsic tissue regeneration potential. Third, both the postoperative period after treatment and the sample size may have been too brief/small to reveal significant differences among experimental groups, although these parameters are in line with other published studies. Finally, technical limitations in bone histology affected the number of samples used for the histological assessment of bone regeneration and the persistence analysis of allogeneic MSCs in the defect site, thus limiting the statistical significance of the data obtained.

## 5. Conclusions

Our study demonstrates that all the osteogenic TEPs tested promote bone regeneration in a skeletally immature juvenile ovine model of cylindrical bone defects after 6 and 12 weeks of treatment and suggests that the addition of allogeneic WJ-MSCs and PRO-DENSE^TM^ to the TEP formulation could have a positive effect on the clinical outcome. However, we did not see a clear and definitive effect across all study groups. This fact manifest the high influence that different factors, including the choice of a specific animal model, the potency of the MSC batch, and its interaction with the recipient’s microenvironment, could have on the effectiveness of MSCs and, therefore, on the successful translation of innovative investigational therapies from the bench to the bedside. Our results are a vivid reflection of the variability observed in clinical trials and should be considered in the development programme of novel bone tissue engineering therapies using WJ-MSCs, not only for ON but also for other clinical issues requiring bone regeneration. Going further, a lot of preclinical data comprising the benefits of the use of MSCs for multiple conditions can be found in the literature, especially when using *in vitro* models or small-animal models. However, in terms of clinical efficacy, there is still a high degree of variability, and the successful clinical outcome of MSCs cannot be reliably predicted. As a consequence, a correlation between preclinical and clinical results and the number of clinically approved MSC-based ATMPs does not exist. Despite this, MSC-based therapies have been demonstrated as safe with independence of the dose, the route of administration, and the clinical condition, and this fact should be an impetus to encourage the development of more clinical trials in which MSCs do not constitute the last option for few severe patients who do not respond to other treatment arms. In this way, a substantial amount of clinical data would be generated, allowing the identification of markers that could predict the clinical efficacy of a specific MSC-based treatment in a particular group of patients. The impact of mismatch in the immunogenetic background between donor and recipient also deserves further investigation.

## Figures and Tables

**Table 1:** Composition of the experimental groups and treatments.

Group	Number of Treated Sheep (*n* = 16)	Number of Bone Defects (*n* = 64)		Treatment
		6 Weeks (*n* = 32)	12 Weeks (*n* = 32)	
**1**	4 (2 ♂ + 2 ♀)	**8** (4 femurs + 4 tibias)	**8** (4 femurs + 4 tibias)	Biomaterial + acellular hydrogel
**2**	**4** (2 ♂ + 2 ♀)	**8** (4 femurs + 4 tibias)	**8** (4 femurs + 4 tibias)	Biomaterial + cellular hydrogel (3 × 10^6^ mCherry-WJ-MSCs)
**3**	**4** (2 ♂ + 2 ♀)	**8** (4 femurs + 4 tibias)	**8** (4 femurs + 4 tibias)	Acellular hydrogel
**4**	**4** (2 ♂ + 2 ♀)	**8** (4 femurs + 4 tibias)	**8** (4 femurs + 4 tibias)	Cellular hydrogel (3 × 10^6^ mCherry-WJ-MSCs)

The term “Biomaterial” refers to PRO-DENSE^TM^_,_ whereas the term “hydrogel” applies to a mix composed of plasmalyte, hyaluronic acid, fibrinogen, and thrombin. ♂, male; ♀, female.

## Data Availability

The data that support the findings of this study are available from the corresponding authors, R.-C.P. and J.V., upon reasonable request.

## Supplementary Materials

The following supporting information can be downloaded at: https://www.mdpi.com/article/10.3390/cells14120862/s1, Figure S1: Effect of bone type and sex on bone regeneration; Table S1: Histological scoring system for the semi-quantitative evaluation of bone regeneration [61,62,63,64,65]; Table S2: Main findings recorded during the surgical interventions, clinical follow-up, and macroscopic necropsies; Table S3: Body weight and body weight variation.
