# Supplementary material for: Preclinical Assessment in Juvenile Sheep of an Allogeneic Bone Tissue Engineering Product with Wharton’s Jelly Mesenchymal Stromal Cells
Source: Cells. 2025 Jun 7;14(12):862. doi: 10.3390/cells14120862 (PMC12191425; doi:10.3390/cells14120862)
Supplement: Supplementary file 1 [file cells-14-00862-s001.zip › cells-3669258-supplementary.pdf]

SUPPLEMENTARY MATERIAL

**Construct integration**	
Not observed	0
Presence of osteoid tissue or new bone trabeculae in 1 of the 3 edges of the defect	1
Presence of osteoid tissue or new bone trabeculae in 2 of the 3 edges of the defect	2
Presence of osteoid tissue or new bone trabeculae in 3 of the 3 edges of the defect	3
**Newly formed tissue other than bone**	
Not observed	0
Non-mineralized loose connective tissue	1
Non-mineralized dense connective tissue	2
Cartilage	3
**Osteogenesis in the central area**	
Not observed	0
New bone trabeculae in one isolated area	1
New bone trabeculae in 2 or 3 different areas	2
New bone trabeculae distributed throughout the defect	3
**Mineralization of the new bone trabeculae**	
No newly formed trabeculae	0
Non-mineralized fibroreticular bone trabeculae	1
Trabeculae of mineralized lamellar bone	2
**Maximum Score**	**11**

Supplementary Table S1. Histological scoring system for the semi-quantitative evaluation of bone regeneration. The score table was defined according to the defect characteristics and the structures observed in the histological sections. The evaluated parameters were selected according to published studies [61–65].

Animal ID	Group	Observations
7519-O-05	3	Surgery 1: transfixed femur.<br>Unexpected death. Necropsy: presence of colitis, external heart petechiae, and dark urine.
7519-O-07	3	Surgery 2: infection of the femoral wound, suppuration, fever.<br>Necropsy: encapsulated pneumonia, lung fibrosis, and pericarditis.
7519-O-10	4	Necropsy: pulmonary oedema, enlarged lymph nodes, presence of fluid in the pericardium, and mild ascites.
7519-O-13	1	Surgery 1: transfixed tibia. Rupture of femoral synovial capsule.<br>Surgery 2: rupture of femoral synovial capsule.
7519-O-14	3	Surgery 2: haematoma and inflammation around the wound.
7519-O-15	2	Surgery 2: transfixed tibia.<br>Necropsy: pneumonia and enlarged lymph nodes.
7519-O-18	4	Surgery 2: granuloma in the femoral defect. Intensive cures.

Supplementary Table S2. Main findings recorded during the surgical interventions, clinical follow-up, and macroscopic necropsies. The observations related with surgeries were directly attributable to surgical interventions. In the case of transfixed bones, this fact was taken into account in the pertinent analysis. The rest of clinical findings were transitory, did not alter the course of the study, and had no impact on the results obtained. None of the findings recorded during the macroscopic necropsies can be attributed to the administration of the TEPs.

	**Weeks**	**Group 1**	**Group 2**	**Group 3**	**Group 4**
**Body Weight (Kg)**	**0**	42.5 ± 12.2	43.6 ± 12.0	44.8 ± 6.4	42.6 ± 9.7
	**2**	43.2 ± 12.7	45.3 ± 11.1	45.0 ± 7.6	48.0 ± 6.5
	**6**	46.8 ± 13.7	51.0 ± 15.2	47.0 ± 8.1	46.8 ± 10.7
	**8**	48.4 ± 13.3	52.1 ± 15.4	46.8 ± 8.4	46.5 ± 15.4
	**12**	53.9 ± 12.5	57.4 ± 15.5	50.5 ± 8.3	52.2 ± 12.8
**Body Weight Variation (%)**	**2**	1.3 ± 2.5	4.4 ± 4.4	0.3 ± 3.7	1.9 ± 2.8
	**6**	9.8 ± 2.4	16.4 ± 7.7	4.7 ± 4.2	10.0 ± 7.8
	**8**	14.1 ± 3.8	19.0 ± 7.1	4.1 ± 6.8	14.7 ± 11.7
	**12**	28.4 ± 7.5	32.1 ± 13.1	12.8 ± 6.9	22.6 ± 10.4

Supplementary Table S3. Body weight and body weight variation. Results indicate mean ± standard deviation. Non-significant differences were found between groups (Kruskal-Wallis test with posterior Dunn's multiple comparison test).

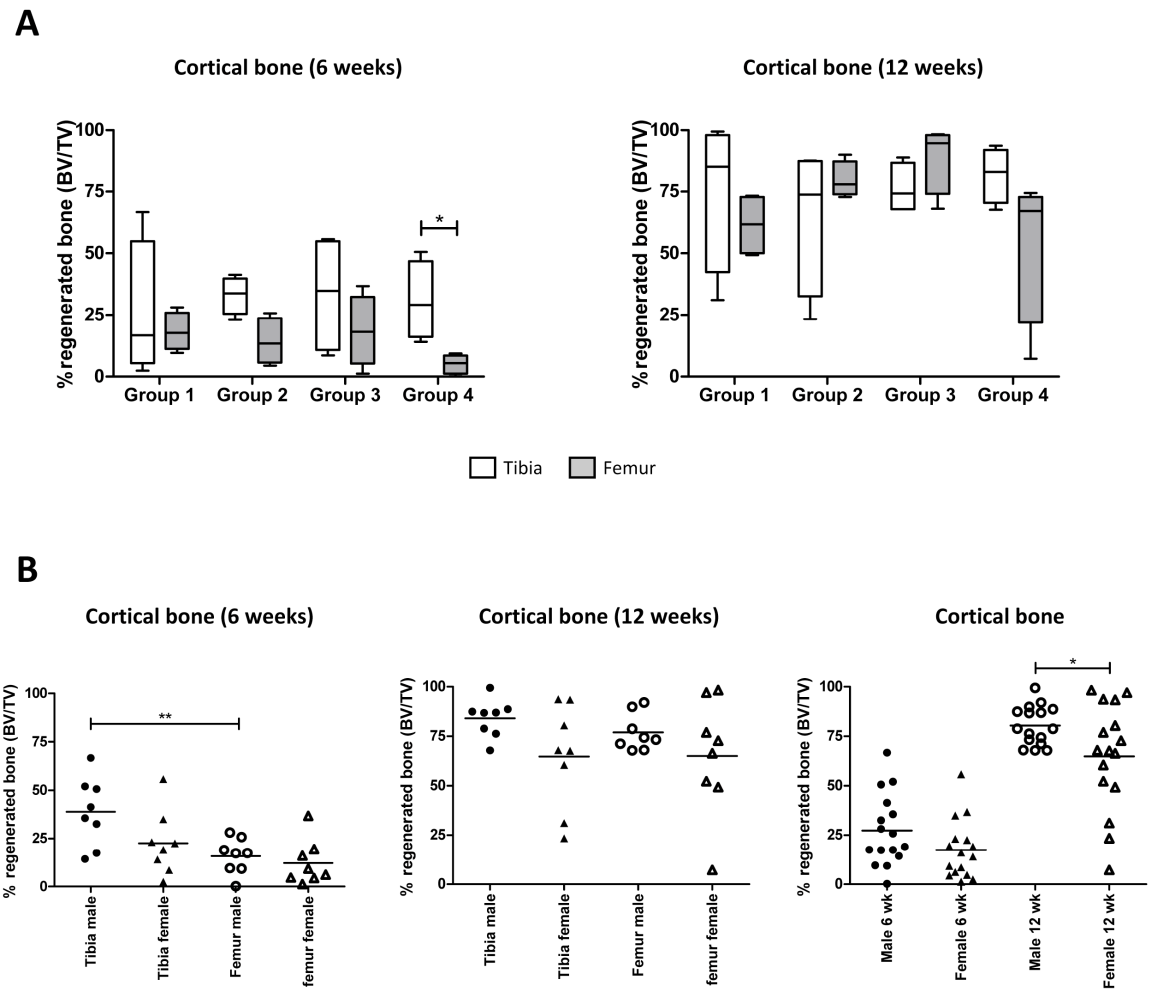

Supplementary Figure S1. Effect of bone type and sex on bone regeneration. (**A**) Quantification of cortical bone regeneration in the defect area in tibia and femur at 6 and 12 weeks after treatment. Whiskers indicate Min to Max values. Statistical significance was set at $p < 0.05$ (*) (Mann-Whitney test). (**B**) Effect of sex on cortical bone regeneration in tibia and femur at 6 and 12 weeks after treatment. Horizontal lines represent the mean. Statistical significance was set at $p < 0.05$ (*) and $p < 0.01$ (**) (Unpaired t-test). BV/TV, bone volume/total volume in the defect area. Wk, weeks.

SUPPLEMENTARY REFERENCES

61. Udehiya, R.K.; Amarpal; Aithal, H.P.; Kinjavdekar, P.; Pawde, A.M.; Singh, R. Comparison of autogenic and allogenic bone marrow derived mesenchymal stem cells for repair of segmental bone defects in rabbits. Res. Vet. Sci. **2013**, *94*, 743–752, doi: 10.1016/j.rvsc.2013.01.011.
62. Pilichi, S.; Rocca, S.; Pool, R.R.; Dattena, M.; Masala, G.; Mara, L.; Sanna, D.; Casu, S.; Manunta, M.L.; Manunta, A.; et al. Treatment with embryonic stem-like cells into osteochondral defects in sheep femoral condyles. *BMC Vet. Res.* **2014**, *10*, 301, doi: 10.1186/s12917-014-0301-9.
63. Lucaciu, O.; Gheban, D.; Soriţau, O.; Băciuţ, M.; Câmpian, R.S.; Băciuţ, G. Comparative assessment of bone regeneration by histometry and a histological scoring system. *Rev. Rom. Med. Lab.* **2015**, *23*, 31–45.
64. Han, Z.; Bhavsar, M.; Leppik, L.; Oliveira, K.M.C.; Barker J.H. Histological Scoring Method to Assess Bone Healing in Critical Size Bone Defect Models. *Tissue Eng.Part C Met.* **2018**, *24*, 272–279.
65. Zhao, L; Zhao, J; Yu, J.J; Zhang, C. Irregular Bone Defect Repair Using Tissue-Engineered Periosteum in a Rabbit Model. *Tissue Eng. Regen Med.* **2020**, *17*, 717–727.
